# *In vivo* analysis of glaucoma-related features within the optic nerve head using enhanced depth imaging optical coherence tomography

**DOI:** 10.1371/journal.pone.0180128

**Published:** 2017-07-21

**Authors:** Tiago S. Prata, Flavio S. Lopes, Vitor G. Prado, Izabela Almeida, Igor Matsubara, Syril Dorairaj, Rafael L. Furlanetto, Roberto M. Vessani, Augusto Paranhos

**Affiliations:** 1 Glaucoma Service, Department of Ophthalmology, Federal University of São Paulo, São Paulo, Brazil; 2 Glaucoma Unit, Hospital Medicina dos Olhos, Osasco, Brazil; 3 Department of Ophthalmology, Mayo Clinic, Jacksonville, United States of America; Bascom Palmer Eye Institute, UNITED STATES

## Abstract

Structural differences between optic nerve head (ONH) parameters in glaucomatous and non-glaucomatous eyes has been documented, however the association between such parameters in patients with different disease stages is yet to be elucidated. We investigated the relationship between different laminar and prelaminar ONH structures using enhanced depth imaging spectral-domain optical coherence tomography (EDI OCT) in a population with and without glaucoma. In this observational case-control study, we prospectively enrolled healthy individuals and glaucomatous patients with different disease stages. All participants underwent EDI OCT imaging (Heidelberg Engineering). Following ONH parameters were measured on serial vertical B-scans by two examiners masked to patient’s clinical data: lamina cribrosa (LC) and prelaminar neural tissue (PLNT) thicknesses, Bruch’s membrane opening (BMO) and cup depth. Associations between cup depth, and laminar and prelaminar parameters were evaluated controlling for possible confounding factors such as axial length and disc size. Sixty-four eyes of 64 patients were included (30 with glaucoma and 34 controls). Eyes with glaucoma had significantly lower mean LC and PLNT thickness, and greater mean cup depth than controls (p<0.01). There was a significant negative association between PLNT thickness and cup depth in glaucomatous eyes (R^2^ = 0.158, p = 0.029). In addition, LC thickness correlated significantly with cup depth (R^2^ = 0.135, p = 0.045). Eyes with thinner LCs presented deeper cups. Overall, cup depth and BMO had the best and LC thickness had the worst intraobserver and interobserver reproducibility grading. In conclusion, significant associations were seen between cup depth, LC and PLNT thickness. Eyes with deeper cups not only had less neural tissue, but also thinner LCs, independent of disc size and axial length. Best reproducibility was found for prelaminar parameters compared to deeper ONH structures.

## Introduction

The lamina cribrosa (LC) within the scleral canal in the optic nerve head (ONH) is thought to be the primary site of axonal insult in glaucoma [1–3]. It is of interest in glaucoma to understand the anatomy of the LC and surrounding tissues and how they change with aging and disease. LC has been investigated using a variety of methods, including histologic section [4,5], detergent digestion of surrounding tissues [6,7] and finite element modelling [8,9].

In human glaucomatous eyes, LC alterations may include posterior deformation [7,10], compression and thinning [10,11], molecular changes in astrocytes and other glial cells [12,13], laminar beam and pore size alterations [14] and posterior migration of both the anterior and posterior laminar insertions from the sclera toward and sometimes into the pial sheath [15].

Previous reports have suggested that spectral domain (SD) optical coherence tomography (OCT) ONH imaging can visualize the LC *in vivo* [16–18]. A relatively new approach to SD-OCT, known as enhanced depth imaging (EDI) OCT, has been shown to improve visibility of deep ONH structures when compared with conventional SD-OCT [19–22]. Even though glaucomatous changes of different ONH parameters have been documented with EDI OCT, the relationship between such parameters in patients with different disease stages is yet to be elucidated, i.e. which ONH parameter has a greater influence on disc cupping in eyes with glaucoma—prelaminar neural tissue loss or LC thinning? The main goal of this study (our primary outcome) was to investigate the relationship between optic disc cupping (cup depth) and lamina cribrosa and prelaminar neural tissue thickness using the EDI modality of SD-OCT in a population with and without glaucoma. As secondary outcomes we determined the interobserver and intraobserver reproducibility of the analyzed EDI OCT parameters.

## Methods

This observational case-control study was conducted at the Department of Ophthalmology of the Federal University of São Paulo. The study protocol was approved by the Institutional Review Board of the Federal University of São Paulo and adhered to the tenets of the Declaration of Helsinki. Informed verbal consent was obtained from all subjects prior to enrollment and examination.

### Participants

We prospectively enrolled consecutive glaucomatous patients with a wide range of disease stages (glaucomatous optic neuropathy (GON) and reproducible visual field (VF) defect) and healthy individuals. All persons gave their informed verbal consent prior to their inclusion in the study. Initially, all participants underwent a complete eye examination. Exclusion criteria for both groups were age <18 years, previous posterior segment intraocular surgery, ocular trauma, significant media opacity, EDI OCT images of poor quality, inability to perform the exams, and ocular diseases other than glaucoma.

Characteristic GON was defined as a vertical cup-to-disc ratio (VCDR) ≥0.6, asymmetry of VCDR≥0.2 between eyes, presence of localized or diffuse peripapillary retinal nerve fiber layer (pRNFL) defects and/or neuroretinal rim defects in the absence of any other abnormalities that could explain such findings [23,24]. A glaucomatous VF defect in the standard automated perimetry (Humphrey SITA—Standard 24–2, Carl Zeiss Meditec, Dublin, CA) was defined as three or more points in clusters with a probability of <5% (excluding those on the edge of the field or directly above and below the blind spot) on the pattern deviation plot, a pattern standard deviation index with a probability of <5%, or a glaucoma hemifield test with results outside the normal limits.

To be included as controls, healthy individuals required normal VF testing and normal appearance of the optic disc defined as a VCDR <0.6, without localized or diffuse pRNFL defects and/or neuroretinal rim defects and untreated IOP <21mmHg [23].

### Imaging and data collection

EDI OCT imaging (SD-OCT; Spectralis, Wavelength: 870nm; Heidelberg Engineering Co., Heidelberg, Germany) was performed for both eyes of each participant. Serial vertical B-scan images of the ONH were obtained using a method previously described [19,21]. The OCT device was set to image a 15° (vertically) × 10° (horizontally) rectangle (vertical scans) centered on the optic disc with a 30° retinal window. The EDI mode of the Spectralis SD-OCT automatically places the OCT reference plane toward the bottom of the OCT acquisition screen, switching the zero delay to the bottom of the OCT screen, without the need of image inversion. This allows an enhancement of the image of deeper layers of ONH. This rectangle was scanned with 97 sections, with an interval between adjacent sections of approximately 30 μm and each section had 100 OCT frames averaged.

**Fig 1:**
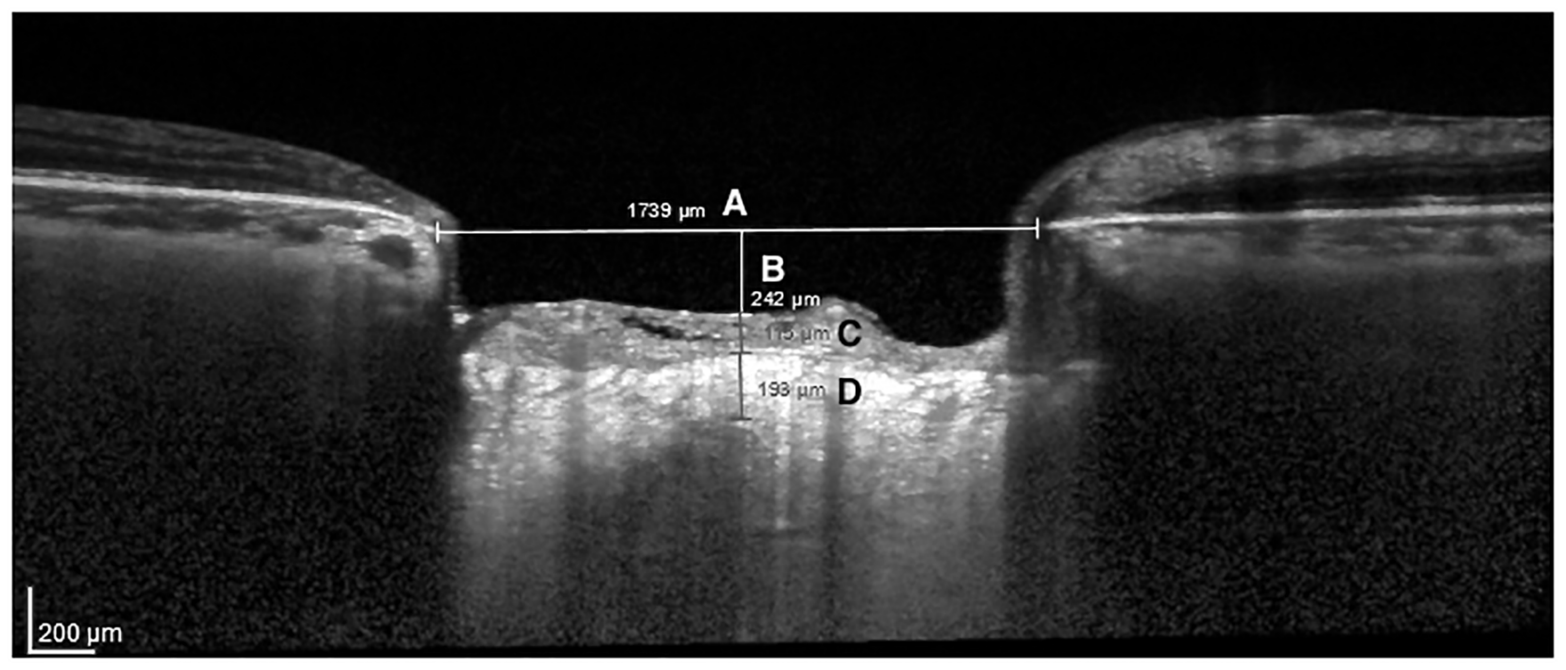
EDI OCT B-scan image showing each optic nerve head parameter evaluated in the study. A: Bruch’s membrane opening; B: Cup depth; C: Prelaminar neural tissue thickness; D: Lamina cribrosa thickness.

The following ONH parameters were measured in EDI OCT vertical B-scans by two experienced examiners masked to patients clinical data: LC thickness and prelaminar neural tissue thickness, Bruch’s membrane opening (BMO) and cup depth (Fig 1). Only good quality images were considered for analysis, presenting at least a signal-to-noise ratio greater than 25 dB as suggested by the OCT device manufacturer. To enhance LC visibility at the EDI OCT scans, image contrast was increased to the maximum level and image colours were switched (black or white) as needed using the device software. The line connecting Bruch’s membrane edges was used as a reference plane for all depth measurements [25]. The selected parameters were measured in the B-scans as close to the horizontal center of the ONH, using the built-in software calipers (unit of measurement: microns). If the chosen image presented vascular shadows that compromised LC or prelaminar tissues visualization, we used the closest temporal B-scan that did not present such artifacts (approximately 30 μm apart). Therefore, LC and prelaminar tissue thicknesses were measured as close to the vertical center of the ONH as possible. The LC thickness was defined as the distance between the anterior and posterior borders of the highly reflective region at the vertical center of the ONH in the vertical EDI OCT cross-sectional B-scans [22]. The prelaminar neural tissue thickness was defined as the perpendicular distance between the anterior prelaminar neural tissue surface and the anterior surface of the LC. Cup depth was defined as the perpendicular distance from the reference line to the anterior prelaminar neural tissue surface. Whenever both eyes were eligible, one was randomly selected for analysis.

### Statistical analysis

Descriptive analysis was used to present demographic and clinical data. D’Agostino–Pearson’s test was performed to determine whether the data had a normal distribution or not. Descriptive statistics included mean and standard deviation for normally distributed variables and median, quartiles for those non-normally distributed. Independent samples t-test was used to compare continuous normally distributed variables between groups, while the Mann–Whitney test was used to compare those non-normally distributed. Categorical data were compared using chi-square test. Scatter plots and regression lines were constructed to investigate possible associations between cup depth and laminar and prelaminar neural tissue thicknesses. Multiple regression analysis was used to account for possible confounding factors, such as disc size (disc size was based on EDI OCT BMO measurements) and axial length, as these anatomical parameters could be related to the main variables being investigated in the study.

To examine the inter-observer reproducibility, two independent examiners (TSP and RMV) assessed a random subset of the study images (n = 19 patients). To examine the intraobserver repeatability, each examiner evaluated twice this same set of images. For each EDI OCT parameter, repeatability and reproducibility were assessed using within-subject standard deviation (Sw) and coefficient of variation (COV; 100%×Sw/overall mean), repeatability coefficient (RC; 1.96x√(2S2w) or 2.77 Sw), intraclass correlation coefficient (ICC; measurement of inter-rater reliability; set for absolute agreement) and Kappa values (inter-rater agreement). Computerized analysis was performed using MedCalc software (MedCalc Inc., Mariakerke, Belgium). The alpha level (type I error) was set at 0.05.

## Results

From the 72 patients (72 eyes) initially examined, 64 eyes of 64 patients were included in the study and considered for analysis (30 with glaucoma and 34 controls). Considering the 30 glaucomatous patients, most of them had primary open-angle glaucoma (63%). Other diagnoses included primary angle-closure glaucoma (27%), secondary glaucomas (7%) and pigmentary glaucoma (3%). The average number of glaucoma medications was 1.6±1.1, and 33.3% of the glaucomatous patients had undergone a glaucoma procedure (80% incisional surgery and 20% laser surgery). Among the 8 eyes that were not considered for analysis due to poor quality imaging or due to impossibility of delineating the posterior border of the LC properly, 3 were from the glaucoma group and 5 were controls. Baseline characteristics of study patients are provided in Table 1.

**Table 1:** Demographic and ocular characteristics of study patients.

Variables*	Control Group (n = 34)	Glaucoma Group (n = 30)	P value
Age (years)	60.7±10.7	63.4±12.6	0.36
Gender (F/M)	22/12	10/20	0.02
Race (W/O)	18/16	16/14	0.83
IOP (mmHg)	14.2±2.5	14.4±3	0.76
CCT (μm)	547±31	511±37.8	<0.01
Axial length (mm)	23.1±0.9	23±0.9	0.76
Vertical Cup-to-disc ratio (mm2)	0.34±0.1	0.84± 0.1	<0.01
Average RNFL thickness (μm)	101.1±14.3	65.8±13.3	<0.01
VFMD (dB)	-1±0.9	-10.4±7	<0.01
VFI (%)	99±1	72.8±23.5	<0.01

F, female; M, male; W, White; O, others; IOP, intraocular pressure; CCT, central corneal thickness; VFMD, visual field mean deviation; VFI, visual field index.

*Data are given as mean±standard deviation whenever indicated.

Table 2 provides in detail all ONH measurements obtained through EDI OCT imaging. When comparing each ONH parameter between eyes with and without glaucoma, we found significantly lower mean LC and prelaminar neural tissue thickness values (p<0.01), and greater mean cup depth values in those with glaucoma (p<0.01).

**Table 2:** Optic nerve head parameters of study patients.

Parameters*	Control Group (n = 34)	Glaucoma Group (n = 30)	P value
PLNTT (μm)	202.5 (114.5, 414.5)	94.5 (67, 124.5)	<0.01
BMO (μm)	1497.5±175.2	1592.6±201.3	0.05
Cup depth (μm)	119.5 (0, 241	391.5 (287, 455)	<0.01
LCT (μm)	165.3±31.5	135.9±30.8	<0.01

PLNTT, prelaminar neural tissue thicknesses; BMO, Bruch’s membrane opening; LCT, lamina cribrosa thickness.

*Normally distributed variables represented by mean±standard deviation; non-normally distributed variables represented by median (first quartile, third quartile).

Multiple regression analysis revealed a significant negative association between prelaminar neural tissue thickness and cup depth in glaucomatous eyes (R^2^ = 0.158, p = 0.029), in which the eyes with thinner prelaminar neural tissue had deeper cups. In addition, LC thickness correlated significantly with cup depth (R^2^ = 0.135, p = 0.045), eyes with thinner LCs presenting deeper cups. Age, race, disc size, axial length and central corneal thickness were not significant in this model (p≥0.08).

**Fig 2:**
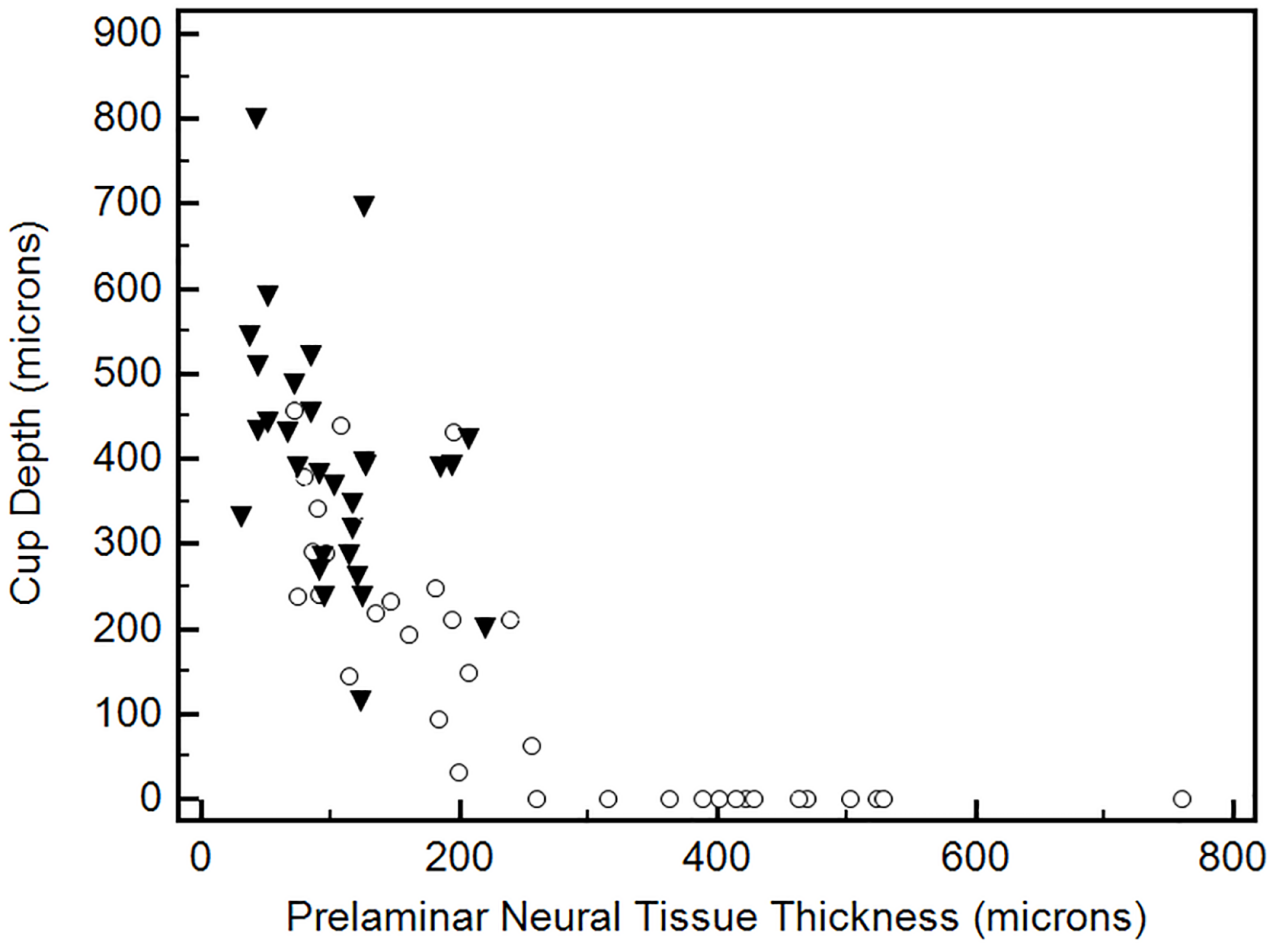
Scatter plot of prelaminar neural tissue thickness against cup depth values for the entire study population (black marks, glaucoma patients; white marks, controls).

**Fig 3:**
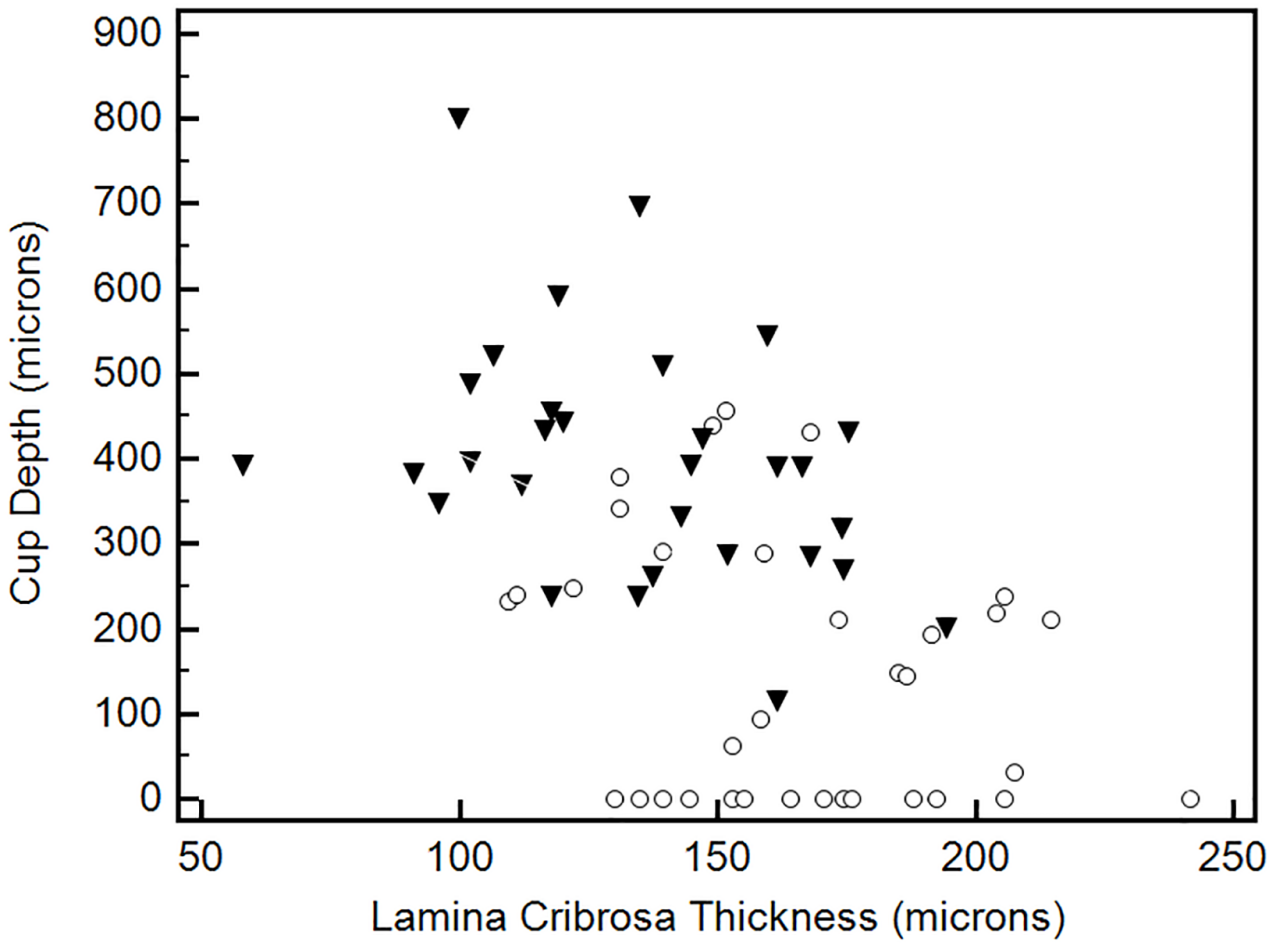
Scatter plot of lamina cribrosa thickness against cup depth values for the entire study population (black marks, glaucoma patients; white marks, controls).

The association between laminar and prelaminar neural tissue thickness with cup depth is shown in Figs 2 and 3. These two plots included both glaucomatous and control eyes to provide a value distribution for the entire study population. In Fig 2, one can note a non-linear correlation between prelaminar neural tissue thickness and cup depth. Cup depth values are homogeneously low until prelaminar neural tissue thickness reaches approximately 250 microns. In fact, above this cut-off value, there are no glaucomatous eyes. When we look below this cut-off value, cup depth values start to increase, and a predominance of eyes with glaucoma is observed. When it comes to the relationship between LC thickness and cup depth, one can note a more linear correlation, as shown in Fig 3. Nevertheless, a significant overlap between glaucomatous and control eyes is observed regarding LC thickness values.

In general, most ONH parameters assessed by EDI OCT showed good repeatability (Sw range, 4.7–19.1 μm; COV range, 0.55%– 7.3%; RC range, 13.1–52.9 μm). Judged by the COV, BMO had the best and LC thickness had the worst intraobserver repeatability. Judged by the ICC (range, 0.70–0.99) and kappa values (range, 0.39–0.78), BMO and cup depth had the best and LC thickness had the worst interobserver reliability and inter-rater agreement.

## Discussion

In this *in vivo* imaging study, we demonstrated significant associations between cup depth and prelaminar neural tissue and laminar thickness. Eyes with deeper cups had not only less neural tissue, but also thinner LCs, independent of disc size and axial length. In addition, while evaluating the reproducibility of each EDI OCT parameter, we found good results for most.

One of the most important requisites of any imaging device is the reproducibility of its findings or measurements as it is essential to distinguish whether changes observed during follow-up examination are related to the test variability or indicate a real progression of the disease. Based on non-automated measurements performed by experienced examiners, most ONH parameters assessed in our study showed good intra-observer and inter-observer reproducibility. Although relatively good reproducibility results were found for LC thickness, it showed the highest measurement variability and the worst inter-rater agreement among all EDI OCT parameters evaluated. There are scant data in the literature regarding the reproducibility of EDI OCT measurements. Evaluating eyes with and without glaucoma in a recent study, Park et al [22] reported good reproducibility indices based on non-automated measurements. In their study the LC thickness was the only parameter evaluated. To the best of our knowledge, the present study is the first to compare the reproducibility of different EDI OCT parameters.

The key findings of our study were the significant associations found between disc cupping (cup depth), and laminar and prelaminar parameters. Deeper cups were associated with thinner LC and prelaminar neural tissue, independent of disc size and axial length. Several previous studies have documented LC characteristics using EDI and Swept-Source OCT [20–26]. However, most of them did not evaluate anatomical relationships between ONH parameters in glaucoma, and focused solely on the structural differences between glaucomatous and non-glaucomatous eyes. In addition, these previous studies also focused on parameters not evaluated in the present study, such as posterior laminar displacement, laminar defects and disinsertions [25,27]. Park et al [22] also reported thinner LC in glaucomatous eyes compared to normal controls. Interestingly, in their study, normal-tension glaucoma eyes had a thinner LC than those with primary open-angle glaucoma eyes. The presence of a disc haemorrhage was associated with an even thinner LC. The authors hypothesized that this structural weakness at the LC may cause failure in capillary-containing laminar beams, which may result in the mechanical rupture of prelaminar capillaries, leading to the disc haemorrhage. Regarding other laminar findings, another EDI OCT study evaluating the LC position in eyes with and without glaucoma found that the anterior surface of the LC was more posteriorly placed in those with the disease, which is consistent with the concept of posterior migration of the LC in glaucoma [25]. However, they did not evaluate LC thickness, which limited their findings. Posteriorization of the anterior surface of the LC may suggest LC compression (thinning), posterior migration or both, depending on the position of the posterior LC surface. In our study, we were able to assess LC thickness and demonstrate that while it is thinner in glaucomatous eyes, it also appears to become thinner as disc cupping progresses.

We believe it is important to discuss the clinical implications of our main findings. The high-technology imaging devices introduced into glaucoma practice over the last decades are intended to detect the earliest structural changes and to support ophthalmologists in clinical decision-making. Moreover, in order to better understand the features related to ONH insult in glaucoma, researchers have been using some of these imaging devices to identify structural alterations in glaucomatous eyes [16–25]. In this study, using EDI OCT technology, we were able to quantify and correlate (*in vivo*) different parameters within the ONH in glaucomatous and healthy eyes. As the primary site of axonal insult in glaucoma [1–3], it is of interest to understand how the LC and surrounding tissues change with the disease. Although based on cross-sectional analysis, our data support the hypothesis that the process of disc cupping in glaucoma is not only associated with prelaminar neural tissue loss (and posterior laminar displacement, as documented in previous studies), but also with progressive laminar thinning. Whether the latter takes place simultaneously or before the former (as a possible predisposing factor) still needs to be determined in longitudinal analysis. It is very important to emphasize that the abovementioned mechanisms and/or processes are just one of the possible reasons to explain the relationships between disc cupping and laminar thinning and neural tissue loss, which are the main findings of our study. Therefore, at the present moment, cause and effect relationships cannot be extrapolated based on our findings.

Some specific characteristics and limitations of our study should be considered while interpreting its results. First, glaucomatous eyes in our study had thinner corneas than controls. Previous studies have shown a significant association between CCT and the severity of glaucomatous damage; eyes with thinner corneas having more advanced glaucomatous damage [28]. The fact that we have patients with moderate to advanced disease stage on average in our study possibly explains the CCT difference between groups. It is important to emphasize that this fact probably did not influence our results, as previous studies have not found any relationship between CCT and LC parameters [29,30]. Second, our results apply solely to this specific study population, and should not be extrapolated to glaucomatous patients with different characteristics. Third, the ONH parameters significantly associated with cup depth in these glaucomatous eyes with various disease stages had a weak coefficient of determination (R^2^ range, 0.13–0.16). Therefore, these associations accounted for only part of cup depth variations observed in these patients. Although statistically significant, their clinical relevance and definitive role during the glaucomatous insult to the ONH is still to be determined. In addition, we believe that these findings suggest that other factors not evaluated in the present study could be related to optic disc cupping in glaucoma. Fourth, we did not compare each ONH parameter between patients with different types of glaucoma due to the relative small sample size of our study. Finally, the cross-sectional design of our study does not allow the investigation of the chronological sequence of the events observed within the ONH nor cause-effect relationships.

In summary, *in vivo* assessment of ONH structures in healthy and glaucomatous eyes revealed significant associations between cup depth and prelaminar neural tissue and laminar thicknesses. Eyes with deeper cups not only had less neural tissue, but also thinner LCs, independent of disc size and axial length. Although derived from cross-sectional data, we believe these results add to the current knowledge of how the LC and surrounding tissues change with the disease and provide the basis for future longitudinal *in vivo* studies.

## Supporting information

S1 Raw Data Table A and Table B. (XLS)

## References

[1] Burgoyne CF, Downs JC, Bellezza AJ, Suh JK, Hart RT. The optic nerve head as a biomechanical structure: a new paradigm for understanding the role of IOP-related stress and strain in the pathophysiology of glaucomatous optic nerve head damage. Prog Retin Eye Res. 2005; 24(1):39–73. doi: 10.1016/j.preteyeres.2004.06.001 . Epub 2004/11/24. eng. 15555526 10.1016/j.preteyeres.2004.06.001

[2] Quigley HA, Flower RW, Addicks EM, McLeod DS. The mechanism of optic nerve damage in experimental acute intraocular pressure elevation. Invest Ophthalmol Vis Sci. 1980; 19(5):505–17. . Epub 1980/05/01. eng. 6154668

[3] Hernandez MR. The optic nerve head in glaucoma: role of astrocytes in tissue remodeling. Prog Retin Eye Res. 2000; 19(3):297–321. 10749379 10.1016/s1350-9462(99)00017-8

[4] Anderson DR. Ultrastructure of Human and Monkey Lamina Cribrosa and Optic Nerve Head. Arch Ophthalmol. 1969; 82(6):800–14. 4982225 10.1001/archopht.1969.00990020792015

[5] Radius RL, Gonzales M. Anatomy of the Lamina Cribrosa in Human Eyes. Arch Ophthalmol. 1981; 99(12):2159–62. 7030283 10.1001/archopht.1981.03930021035010

[6] Quigley HA. Optic Nerve Damage in Human Glaucoma. Arch Ophthalmol. 1981; 99(4):635 6164357 10.1001/archopht.1981.03930010635009

[7] Quigley HA, Addicks EM. Regional Differences in the Structure of the Lamina Cribrosa and Their Relation to Glaucomatous Optic Nerve Damage. Arch Ophthalmol. 1981; 99(1):137–43. 7458737 10.1001/archopht.1981.03930010139020

[8] Sigal IA, Flanagan JG, Tertinegg I, Ethier CR. Finite Element Modeling of Optic Nerve Head Biomechanics. Investigative Opthalmology & Visual Science. 2004; 45(12):4378. 10.1167/iovs.04-0133 15557446

[9] Downs JC, Roberts MD, Burgoyne CF, Hart RT. Multiscale finite element modeling of the lamina cribrosa microarchitecture in the eye. 2009 Annual International Conference of the IEEE Engineering in Medicine and Biology Society; 2009/09: Institute of Electrical and Electronics Engineers (IEEE); 2009. 10.1109/IEMBS.2009.5332755 PMC3268703 19963817

[10] Jonas JB, Berenshtein E, Holbach L. Anatomic Relationship between Lamina Cribrosa, Intraocular Space, and Cerebrospinal Fluid Space. Investigative Opthalmology & Visual Science. 2003; 44(12):5189. 10.1167/iovs.03-0174 14638716

[11] Quigley HA, Hohman RM, Addicks EM, Massof RW, Green WR. Morphologic Changes in the Lamina Cribrosa Correlated with Neural Loss in Open-Angle Glaucoma. Am J Ophthalmol. 1983; 95(5):673–91. 6846459 10.1016/0002-9394(83)90389-6

[12] Hernandez MR, Andrzejewska WM, Neufeld AH. Changes in the Extracellular Matrix of the Human Optic Nerve Head in Primary Open-Angle Glaucoma. Am J Ophthalmol. 1990; 109(2):180–8. 2405683 10.1016/s0002-9394(14)75984-7

[13] Agapova O. Altered expression of 3α-hydroxysteroid dehydrogenases in human glaucomatous optic nerve head astrocytes. Neurobiol Dis. 2003; 14(1):63–73. 13678667 10.1016/s0969-9961(03)00101-3

[14] Tezel G. Alterations in the morphology of lamina cribrosa pores in glaucomatous eyes. Br J Ophthalmol. 2004; 88(2):251–6. doi: 10.1136/bjo.2003.019281 14736786 10.1136/bjo.2003.019281 PMC1772022

[15] Yang H, Williams G, Downs JC, Sigal IA, Roberts MD, Thompson H, et al Posterior (Outward) Migration of the Lamina Cribrosa and Early Cupping in Monkey Experimental Glaucoma. Investigative Opthalmology & Visual Science. 2011; 52(10):7109. 10.1167/iovs.11-7448 PMC3207714 21715355

[16] Vilupuru AS, Rangaswamy NV, Frishman LJ, Smith EL Iii, Harwerth RS, Roorda A. Adaptive optics scanning laser ophthalmoscopy for in vivo imaging of lamina cribrosa. Journal of the Optical Society of America A. 2007; 24(5):1417. 10.1364/josaa.24.001417 PMC4696388 17429488

[17] Kagemann L, Ishikawa H, Wollstein G, Brennen PM, Townsend KA, Gabriele ML, et al. Ultrahigh-resolution spectral domain optical coherence tomography imaging of the lamina cribrosa. Ophthalmic Surg Lasers Imaging. 2008; 39(4 Suppl):S126–31. . Epub 2008/09/10. eng. 18777881 10.3928/15428877-20080715-07 PMC2908153

[18] Strouthidis NG, Grimm J, Williams GA, Cull GA, Wilson DJ, Burgoyne CF. A comparison of optic nerve head morphology viewed by spectral domain optical coherence tomography and by serial histology. Invest Ophthalmol Vis Sci. 2010; 51(3):1464–74. doi: 10.1167/iovs.09-3984 . Epub 2009/10/31. eng. 19875649 10.1167/iovs.09-3984 PMC2829380

[19] Spaide RF, Koizumi H, Pozonni MC. Enhanced Depth Imaging Spectral-Domain Optical Coherence Tomography. Am J Ophthalmol. 2008; 146(4):496–500. doi: 10.1016/j.ajo.2008.05.032 18639219 10.1016/j.ajo.2008.05.032

[20] Lee EJ, Kim T-W, Weinreb RN, Park KH, Kim SH, Kim DM. Visualization of the Lamina Cribrosa Using Enhanced Depth Imaging Spectral-Domain Optical Coherence Tomography. Am J Ophthalmol. 2011; 152(1):87–95.e1. doi: 10.1016/j.ajo.2011.01.024 21570046 10.1016/j.ajo.2011.01.024

[21] Park SC, De Moraes CGV, Teng CC, Tello C, Liebmann JM, Ritch R. Enhanced Depth Imaging Optical Coherence Tomography of Deep Optic Nerve Complex Structures in Glaucoma. Ophthalmology. 2012; 119(1):3–9. doi: 10.1016/j.ophtha.2011.07.012 21978593 10.1016/j.ophtha.2011.07.012

[22] Park H-YL, Jeon SH, Park CK. Enhanced Depth Imaging Detects Lamina Cribrosa Thickness Differences in Normal Tension Glaucoma and Primary Open-Angle Glaucoma. Ophthalmology. 2012; 119(1):10–20. doi: 10.1016/j.ophtha.2011.07.033 22015382 10.1016/j.ophtha.2011.07.033

[23] Lopes FSS, Dorairaj S, Junqueira DLM, Furlanetto RL, Biteli LG, Prata TS. Analysis of neuroretinal rim distribution and vascular pattern in eyes with presumed large physiological cupping: a comparative study. BMC Ophthalmol. 2014; 14(1). 10.1186/1471-2415-14-72 PMC4047008 24885255

[24] Prata TS, Dorairaj S, Trancoso L, Kanadani FN, Biteli LG, Furlanetto R, et al Eyes with large disc cupping and normal intraocular pressure: using optical coherence tomography to discriminate those with and without glaucoma. Medical hypothesis, discovery and innovation in ophthalmology. 2014; 3(3):91–8. 25741525 PMC4348491

[25] Furlanetto RL, Park SC, Damle UJ, Fernando Sieminski S, Kung Y, Siegal N, et al Posterior Displacement of the Lamina Cribrosa in Glaucoma: In Vivo Interindividual and Intereye Comparisons. Investigative Opthalmology & Visual Science. 2013; 54(7):4836. 10.1167/iovs.12-11530 23778876

[26] Girard MJA, Tun TA, Husain R, Acharyya S, Haaland BA, Wei X, et al Lamina Cribrosa Visibility Using Optical Coherence Tomography: Comparison of Devices and Effects of Image Enhancement Techniques. Invest Ophthalmol Vis Sci. 2015; 56(2):865–74. doi: 10.1167/iovs.14-14903 25593025 10.1167/iovs.14-14903

[27] Sharpe GP, Danthurebandara VM, Vianna JR, Alotaibi N, Hutchison DM, Belliveau AC, et al Optic Disc Hemorrhages and Laminar Disinsertions in Glaucoma. Ophthalmology. 2016; 123(9):1949–56. doi: 10.1016/j.ophtha.2016.06.001 27432205 10.1016/j.ophtha.2016.06.001

[28] Prata TS, Lima VC, Guedes LM, Biteli LG, Teixeira SH, de Moraes CG, et al Association between corneal biomechanical properties and optic nerve head morphology in newly diagnosed glaucoma patients. Clin Exp Ophthalmol. 2012 Sep-Oct;40(7):682–8. doi: 10.1111/j.1442-9071.2012.02790.x 22429725 10.1111/j.1442-9071.2012.02790.x

[29] Jonas JB, Holbach L. Central corneal thickness and thickness of the lamina cribrosa in human eyes. Invest Ophthalmol Vis Sci 2005:46:1275–9. doi: 10.1167/iovs.04-0851 15790890 10.1167/iovs.04-0851

[30] Ren R, Li B, Gao F, Li L, Xu W, Wang N, et al Central corneal thickness, lamina cribrosa and peripapillary scleral histomophometry in non-glaucomatous Chinese eyes. Graefes Arch Clin Exp Ophthalmol 2010; 248:1579–85. doi: 10.1007/s00417-010-1369-y 20495817 10.1007/s00417-010-1369-y

